# Strength of clinical evidence leading to approval of novel cancer medicines in Europe: A systematic review and data synthesis

**DOI:** 10.1002/prp2.816

**Published:** 2021-07-07

**Authors:** Alberto Farina, Federico Moro, Frederick Fasslrinner, Annahita Sedghi, Miluska Bromley, Timo Siepmann

**Affiliations:** ^1^ Division of Healthcare Sciences Center for Clinical Research and Management Education Dresden International University Dresden Germany; ^2^ Medical Affairs Department Celltrion Healthcare Italy srl Milan Italy; ^3^ Laboratory of Acute Brain Injury and Therapeutic Strategies, Department of Neuroscience Istituto di Ricerche Farmacologiche Mario Negri IRCCS Milan Italy; ^4^ Department of Internal Medicine I University Hospital Carl Gustav Carus Technische Universität Dresden Dresden Germany; ^5^ Department of Neurology University Hospital Carl Gustav Carus Technische Universität Dresden Dresden Germany; ^6^ Universidad Cientifica del Sur Lima Peru

**Keywords:** blinding, evidence‐based medicine, anti‐cancer drugs, overall survival, randomized controlled trial, uncontrolled trial

## Abstract

We aimed to evaluate the quality of clinical evidence that substantiated approval of cancer medicines by the European Medicines Agency (EMA) in the last decade. We performed a systematic review and data synthesis of EMA documents in agreement with PRISMA guidelines. We included the European Public Assessment Reports, Summaries of Product Characteristics, and published randomized controlled trials (RCTs) on anti‐cancer drugs approved by EMA from 2010 to 2019, and excluded drugs not indicated for targeting solid or hematological tumors and non‐innovative treatments. We synthesized frequencies of approvals differentiating between unblinded and blinded RCTs with and without overall survival (OS) as a predefined primary outcome measure. We assessed the frequency of post‐approval RCTs for indications without at least one RCT at the time of approval. Of 199 approvals, 159 (80%) were supported by at least one RCT, 63 (32%) by at least one RCT having OS as the primary or co‐primary endpoint, 74 (37%) by at least one blinded RCT, and 30 (15%) by at least one blinded RCT having OS as the primary or co‐primary endpoint. Whereas 40 approvals (20%) were not supported by any RCT and, of those, 9 (22%) were followed by a post‐approval RCT. While the majority of approvals of cancer medicines approved by EMA was supported by at least one RCT, we noted substantial methodological heterogeneity of the studies.

**Clinical trial registration**: PROSPERO registration number CRD42020206669.

## Graphical abstract

Systematic review with the primary objective of describing the frequency of new cancer indications approved by European Medicines Agency (EMA) from 2010 to 2019 and supported by at least one RCT having OS as a primary or co‐primary endpoint. Of 199 approvals, 159 (80%) were supported by at least one RCT, 63 (32%) by at least one RCT having OS as the primary or co‐primary endpoint, 74 (37%) by at least one blinded RCT, and 30 (15%) by at least one blinded RCT having OS as the primary or co‐primary endpoint. Whereas 40 approvals (20%) were not supported by any RCT and, of those, 9 (22%) were followed by a post‐approval RCT. Included studies displayed methodological heterogeneity.

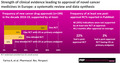

**Abbreviations**
ATC: Anatomical Therapeutic Chemical
CE: comparative evidence
DFS: disease‐free survival
EMA: European Medicines Agency
EPAR: European Public Assessment Report
ESMO‐MCBS: European Society for Medical Oncology Magnitude of Clinical Benefit Scale
FDA: Food and Drug Administration
MCB: meaningful clinical benefit
OS: overall survival
PFS: progression‐free survival
PRISMA: Preferred Reporting for Systematic Reviews and Meta‐analyses
QoL: quality of life
RCT: randomized controlled trial
SPC: Summary of Product Characteristics

## 1 INTRODUCTION

Cancer is the second leading cause of death in the world.^1^ This huge unmet medical need translated in the approval of numerous new drugs by the regulatory agencies in the recent decade.^2^ Current evidence shows that several cancer indications are approved without randomized controlled trials (RCTs) and without overall survival (OS) data as a primary endpoint.^3^, ^4^, ^5^, ^6^, ^7^, ^8^, ^9^ These data are still missing even after years from the approval and a comprehensive overview of the approvals that occurred in the last decade is currently missing.

We hypothesized that the urgency of bringing new therapies to the market may be accompanied by poor clinical evidence, in terms of the quality of study design and endpoints used to measure efficacy. Therefore, the objective of our research is to evaluate, in terms of study design and outcomes, the quality of clinical evidence supporting the approval by the European Medicines Agency (EMA) of new drugs and/or indications for the treatment of cancer in the decade 2010–2019.

Randomized controlled trials are commonly recognized as the gold standard for the evaluation of new therapies, providing the strongest level of evidence and proof of cause–effect relationship thanks to their high internal validity.^10^, ^11^ One of the key strengths of RCTs is the provision of comparative evidence (CE) either versus placebo, active treatment or standard of care. CE represents a fundamental asset for the optimal use of a drug at its entry into clinical practice since despite existing methodological complexities, comparative efficacy evidence should have a formal role in drug licensing decisions.^12^ At the time of first approval, in fact, the comparative profile of benefit and risk represents a safeguard for public health by preventing the use of potentially unsafe or inferior treatments compared to the options already on the market. From an economic point of view, CE allows health technology assessment organizations and payers to make better decisions. From the clinical side, it offers doctors and patients the opportunity to understand which is the safest and effective treatment.

In the field of oncology, key treatment goals should be an improvement of clinically relevant endpoints such as OS, quality of life (QoL), or both.^13^ Other survival measures, including the widely used progression‐free survival (PFS), represent surrogate endpoints. PFS may be biased due to difficulty in measuring progression, use of non‐standardized measurement procedures, informative censoring when patients leave the study without documentation of progression or assessor's expectations in case of open‐label studies. The correlation of PFS with OS may be poor in some settings. If survival after progression is long, for example, longer than 12 months, it may be difficult to show benefit in OS, and the use of PFS may be preferable.^14^ However, if a new treatment offers a clear advantage in terms of OS or QoL, surrogate endpoints are not necessary or should be used as a primary endpoint only in the early stages of clinical development. The usefulness of a treatment that has only demonstrated positive effects on a surrogate endpoint, not clearly correlated with OS, is questionable. Moreover, surrogate endpoints often give an overestimation of benefit and may lead to approval of medicines that only provide a marginal benefit in a real‐world setting.^13^ In addition, PFS does not directly measure how a patient really feels or lives; it provides information on the effects of the intervention on the tumor burden process. Therefore, a significant effect on PFS is not enough to achieve reliable evidence of clinical benefit. The real need of cancer patients is to achieve clinically meaningful beneficial effects on disease‐related symptoms, on ability to carry out normal activities, and on OS.^15^ Thus, it is crucial that new cancer drugs also show their capability to increase in OS, QoL, or ideally both.^16^

On the other hand, there are limitations to conducting RCTs and using OS as the primary endpoint, which in some cases can make their implementation not feasible. For example, RCTs may be limited by economic factors or inaccessibility of rare populations.^17^ Using OS as a primary endpoint requires larger sample sizes and longer follow‐up.^18^ In comparison to OS, PFS and response rate as primary endpoint were associated with an 11‐month (95% CI, 5–17 months) and a 19‐month (95% CI, 13–25 months) reduction in the study durations, respectively.^19^

According to EMA, a randomized design versus a comparator arm should be preferred, although exceptions are admissible depending on the specific study setting. Confirmatory trials should demonstrate that the investigational product provides clinical benefits. Acceptable primary endpoints include OS and PFS/disease‐free survival (DFS). If PFS/DFS is the selected primary endpoint, OS should be reported as a secondary and vice versa. When OS is reported as a secondary endpoint, the estimated treatment effect on OS should ensure that there are no relevant negative effects on this endpoint, in most cases by showing trends toward superiority. In situations where there is a large effect on PFS, or if there is a long‐expected survival after progression, and/or a clearly favorable safety profile, EMA does not mandatorily require precise estimates of OS for approval.^20^

We aimed to evaluate the quality of study design and outcomes reported by studies supporting EMA approval of new drugs and/or indications for the treatment of cancer in the decade 2010–2019. Therefore, we assessed the frequency of new cancer indications supported by at least one randomized and controlled trial having OS as a primary or co‐primary endpoint.

## 2 MATERIALS AND METHODS

### 2.1 Protocol and registration

We performed a systematic review and synthesized analysis. In accordance with the study design, informed patient consent and ethical and regulatory approval are inapplicable. The present work has been conducted in agreement with Preferred Reporting for Systematic Reviews and Meta‐analyses (PRISMA) guidelines,^21^ and the protocol was registered in the PROSPERO International prospective register of systematic reviews (CRD42020206669).

### 2.2 Eligibility criteria

We included all new drugs and/or new indications for the treatment of solid or hematological tumors approved by EMA in the decade 2010–2019, which had an Anatomical Therapeutic Chemical (ATC) “L” classification (antineoplastic and immunomodulating agents).

Criteria for exclusion from the study were: drugs and/or indications not for the treatment of solid or hematological tumors (e.g., supportive therapy, non‐cancer indications, diagnostic or visualization purposes); indications for solid or hematological tumors approved before January 1, 2010 or after December 31, 2019; drugs approved for the first time before January 1, 2007; non‐innovative drugs and/or indications (e.g., biosimilars, generics, new formulations, well‐established use, approvals based on literature data); indication requests withdrawn before their approval.

### 2.3 Information sources and literature search

Medicine‐specific European Public Assessment Report (EPAR) public pages of the EMA website were accessed.^22^ The EPAR EMA database was chosen because it publishes all the clinical data used to request product authorization, with complete and structured information according to a standard format. The clinical data reported in the Summary of Product Characteristics (SPC) were not used because they are limited compared to EPAR. Articles published in the literature were not used because they may be unpublished at the time of drug approval, they report limited data compared to EPAR, and potentially may be affected by publication and reporting bias.

For each drug, the “initial marketing‐authorization documents” and “changes since initial authorization of medicine” in the Assessment history section of each product page were accessed. The first one reports the data used to request the first authorization, the second one reports the data to request the authorization of further indications if any. Data on studies were obtained from the “main studies” section reported in the clinical efficacy chapter of the “assessment reports.” Only the clinical data reported in the aforementioned section were considered in this research.

Only for indications without RCTs (not reported in EPAR) at the time of first approval, a PubMed and SPC search was conducted to verify the presence of post‐approval randomized studies. PubMed Search parameters: “drug name”[title/abstract] AND randomized[title/abstract]; filter: clinical trial. The SPCs currently in force have been consulted in paragraph 5.1.

### 2.4 Study selection

All kinds of clinical studies reported in the main studies section of EPARs were eligible, since the aim of this study was to describe the evidence supporting new cancer indications approved by EMA. For indications without RCTs at the time of first approval, only post‐approval RCTs were selected.

### 2.5 Data extraction

Extraction of data from assessment reports and full‐text articles was performed by two independent reviewers (AF and FM) and data were inserted into a standardized database form (Excel; Microsoft). In case of inconsistencies, the data were double checked by both researchers and disagreements were solved by consensus.

For each indication meeting the inclusion and exclusion criteria, the following data were collected: complete indications, their classification as a solid or hematological tumor indication, and subsequent general classification based on the type of tumor (e.g., breast cancer, prostate cancer, lymphoma, etc.); presence of orphan drug conditions or conditional approval. For studies reported as “main studies” in the clinical efficacy section of “assessment reports” the following data were collected: number; identification code; randomization and control (yes/no); blinding (yes/no); phase; number of patients enrolled; ongoing status (yes/no); narrative description of control if applicable; OS as a primary or co‐primary endpoint (yes/no); non‐OS as a primary or co‐primary endpoint (yes/no); OS as a secondary endpoint (yes/no); non‐OS as a secondary endpoint (yes/no); presence of the study as a main study for other drugs (e.g., in case of combination therapy).

### 2.6 Quantitative data synthesis and statistical analyses

Descriptive statistics of the drugs and indications included in the study were performed using Microsoft Excel. In case multiple studies were reported for a single indication, the strongest/cumulative variable was also extracted, and, in such case, the indication was considered to be supported by RCT if at least one RCT was present. The same criterion was adopted for blinded RCTs, RCTs having OS as a primary endpoint, and blinded RCTs having OS as a primary endpoint. Similarly, the highest study phase was extracted. The status of studies was reported as ongoing if at least one ongoing study was present. Survival was classified either in overall (OS) or all other parameters to measure survival, collectively defined as non‐OS.

The present analysis did not differentiate between the types of treatment (e.g., neo‐adjuvant or adjuvant) or between the stages (e.g., early or metastatic) or variants of the disease.

For post‐approval studies searched through literature and SPC, the following items were collected: time from approval to current search; presence/absence of RCT in the target indication; presence or absence of OS as a primary endpoint.

The risk of bias in individual studies was not evaluated. A descriptive statistic of the collected data was performed, using mean or median where appropriate to summarize continuous parameters and percentage frequency for categorical parameters.

## 3 RESULTS

### 3.1 Indications included in the study

**FIGURE 1:**
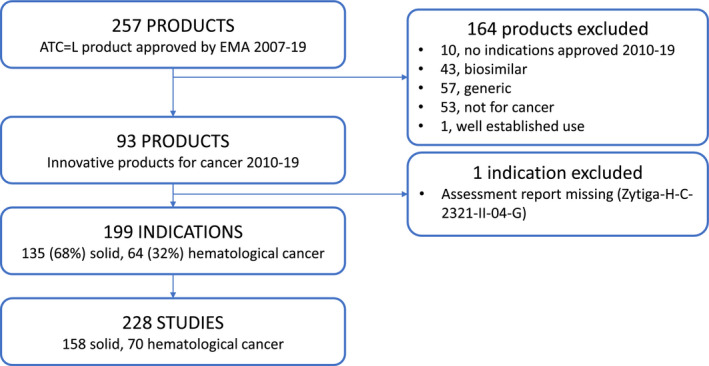
Flowchart showing the number of products and indications included in the study, and the reasons for exclusion. ATC, Anatomical Therapeutic Chemical

Overall, of the 257 products with ATC L classification, 93 were included, for a total of 199 indications and 228 studies (Figure 1). Reasons for exclusion were a date beyond predetermined limits, non‐innovative products (biosimilars, generics, established use) or products not indicated for cancer. For one product only, the EPAR report was not available and it was excluded from the analysis.

### 3.2 Products characteristics

The 93 products included in the study had overall 199 indications approved in the 2010–2019 decade (2.1 mean and 2.0 median indications per product).

Sixty‐four (60.8%), 27 (29%), and 2 (2.2%) products were indicated for solid, hematological, or both types of cancer, respectively. Products indicated for solid, hematological, or both types of cancer presented, respectively: 115 (58%), 62 (31%), 22 (11%) indications; 1.8, 2.3, 11.0 mean indications; 1.0, 2.0; 11.0 median indications.

### 3.3 Indications characteristics

Of the 199 approvals in the past decade included in our analysis, 68% referred to solid tumors and 32% to hematological tumors. The most frequent diagnoses were lung cancer, leukemia, skin cancer, lymphoma, breast cancer, multiple myeloma, renal cell carcinoma, prostate cancer, and colorectal cancer (Table 1). These diseases accounted for approximately 80% of all the indications approved in the past decade. The remaining indications comprised: epithelial ovarian, fallopian tube, or primary peritoneal cancer, gastric cancer, hepatocellular carcinoma, thyroid cancer, urothelial carcinoma, basal cell carcinoma, head and neck cancer, neuroblastoma, neuroendocrine tumors, pancreas adenocarcinoma, soft tissue tumors, hematological malignancies, myelodysplastic syndromes, ovarian cancer, sarcoma, solid tumors.

**TABLE 1:** Summary of indications

	All	Solid cancer	Hematological cancer	Lung cancer	Leukemia	Skin cancer	Lymphoma	Breast cancer	Multiple myeloma	Renal cell carcinoma	Prostate cancer	Colorectal cancer
Indications, No. (%)	199 (100%)	135 (68%)	64 (32%)	31 (15.6%)	29 (14.6%)	22 (11.1%)	19 (9.5%)	18 (9%)	15 (7.5%)	11 (5.5%)	7 (3.5%)	6 (3%)
Main studies, No.	228	158	70	35	29	28	19	23	20	11	7	8
Main studies, mean (median)	1.1 (1)	1.2 (1)	1.1 (1)	1.1 (1)	1 (1)	1.3 (1)	1 (1)	1.3 (1)	1.3 (1)	1 (1)	1 (1)	1.3 (1)
Mean No. of patients	612.5	704.9	417.6	552	337	686	305	1202	768	626	1239	1075
Median No. of patients	495	616	317.5	411	326	673	150	944.5	752.5	723	1199	936
Indications with at least 1 RCT, No. (%)	159 (79.9%)	116 (85.9%)	43 (67%)	22 (71%)	19 (66%)	19 (86%)	10 (53%)	18 (100%)	14 (93%)	11 (100%)	7 (100%)	6 (100%)
Indications with at least 1 blind RCT, No. (%)	74 (37.2%)	63 (46.7%)	11 (17.2%)	7 (22.6%)	3 (10.3%)	14 (63.6%)	4 (21.1%)	11 (61.1%)	4 (26.7%)	1 (9.1%)	6 (85.7%)	4 (66.7%)
Indications with at least 1 study having OS survival as primary endpoint, No. (%)	63 (31.7%)	57 (42.2%)	6 (9.4%)	12 (39%)	6 (21%)	10 (45%)	0 (0%)	4 (22%)	0 (0%)	5 (45%)	5 (71%)	5 (83%)
Indications with at least 1 study having non‐OS as primary endpoint, No. (%)	114 (57.3%)	82 (60.7%)	32 (50%)	16 (52%)	11 (38%)	15 (68%)	8 (42%)	16 (89%)	14 (93%)	10 (91%)	4 (57%)	2 (33%)
Indications with at least 1 study having any survival as primary endpoint, No. (%)	150 (75.4%)	112 (83%)	38 (59.4%)	21 (68%)	17 (59%)	18 (82%)	8 (42%)	17 (94%)	14 (93%)	11 (100%)	7 (100%)	5 (83%)
Indications with at least 1 study having OS survival as secondary endpoint, No. (%)	152 (76.4%)	93 (68.9%)	59 (92.2%)	24 (77%)	25 (86%)	18 (82%)	18 (95%)	14 (78%)	15 (100%)	10 (91%)	1 (14%)	2 (33%)
Indications with at least 1 study having non‐OS as secondary endpoint, No. (%)	136 (68.3%)	91 (67.4%)	45 (70.3%)	22 (71%)	24 (83%)	18 (82%)	15 (79%)	10 (56%)	5 (33%)	7 (64%)	4 (57%)	4 (67%)
Indications with at least 1 study having any survival as primary or secondary endpoint, No. (%)	198 (99.5%)	134 (99.3%)	64 (100%)	31 (100%)	29 (100%)	22 (100%)	19 (100%)	18 (100%)	15 (100%)	11 (100%)	7 (100%)	6 (100%)

Abbreviations: OS, overall survival; RCT, randomized controlled trial.

The number of indications approved each year showed a progressive growing trend from 2010 (4 indications: 2 for solid, 2 for hematological cancer) to 2019 (32 indications: 22 for solid, 10 for hematological cancer).

### 3.4 Key results

**FIGURE 2:**
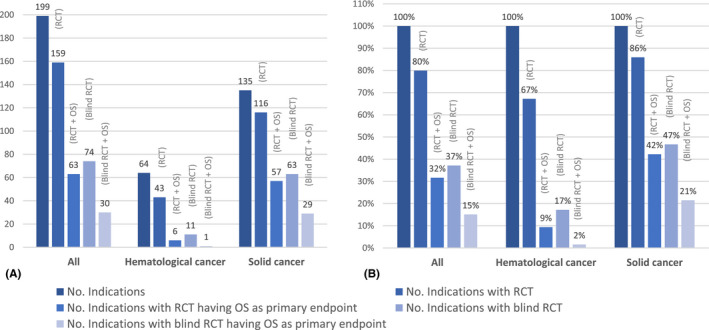
(A) Absolute frequencies of indications, including indications with at least one RCT, at least one RCT with OS as a primary or co‐primary endpoint, at least one blind RCT, at least one blind RCT with OS as a primary or co‐primary endpoint. (B) Relative (%) frequencies per group of indications, including indications with at least one RCT, at least one RCT with OS as a primary or co‐primary endpoint, at least one blind RCT, at least one blind RCT with OS as a primary or co‐primary endpoint. OS, overall survival; RCT, randomized controlled trial

Overall, 159 (80%) of the 199 approved indications were supported by at least one RCT, 63 (32%) by at least one RCT having OS as the primary or co‐primary endpoint, 74 (37%) by at least one blinded RCT, 30 (15%) by at least one blinded RCT having OS as the primary or co‐primary endpoint. Solid tumors and hematological tumors categories presented important differences in the frequency of RCTs (85.2% vs. 67.2%, respectively) and RCTs having OS as a primary or co‐primary endpoint (42.2% vs. 9.4%, respectively; Figure 2A,B). All values decreased further when study blinding and blinding with OS as primary or co‐primary endpoint were considered.

**FIGURE 3:**
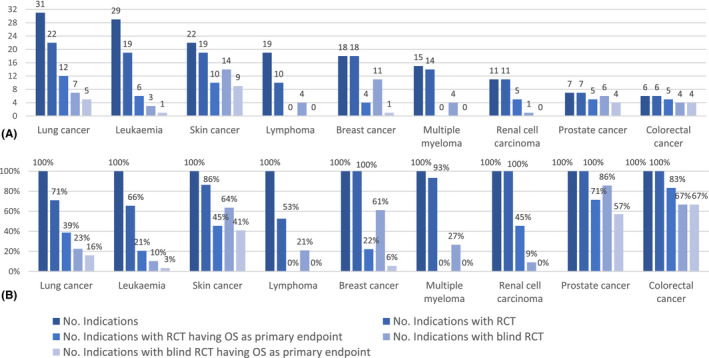
(A) Absolute frequencies of the nine most frequent indications, including indications with at least one RCT, at least one RCT with OS as a primary or co‐primary endpoint, at least one blind RCT, at least one blind RCT with OS as a primary or co‐primary endpoint. (B) Relative (%) frequencies per single indication of the nine most frequent indications, including indications with at least one RCT, at least one RCT with OS as a primary or co‐primary endpoint, at least one blind RCT, at least one blind RCT with OS as a primary or co‐primary endpoint. OS, overall survival; RCT, randomized controlled trial

The nine most frequent indications were supported by at least one RCT with different frequencies, ranging from 53% for lymphoma, to 100% for breast cancer, renal cell carcinoma, prostate cancer, and colorectal cancer. Indications approved by at least one RCT having OS as a primary or co‐primary endpoint ranged from 0% for lymphoma, multiple myeloma, and renal cell carcinoma, to 67% for colorectal cancer (Figure 3A,B).

Forty (20%) indications were not supported by at least one RCT at approval, corresponding to 19 of 135 (14%) solid cancer indications and 21 of 64 (32%) hematological cancer indications. Overall, 44.2 months after approval on average, we identified the presence of a post‐approval RCT reported in PubMed in 22% of cases, and a post‐approval RCT having OS as a primary endpoint in 2% of cases. These frequencies did not match with the data found in the SPC search: We found the presence of a post‐approval RCT in 20% of cases, and a post‐approval RCT having OS as a primary endpoint in 5% of cases (Table 2).

**TABLE 2:** Summary of post‐approval RCTs reported in PubMed and current SPCs for indications not supported by RCTs at the time of approval

	No. of indications	Months since approval (mean)	No. (%) of indications with RCT in PubMed	No. (%) of indications with RCT having OS as a primary endpoint in PubMed	No. (%) of indications with RCT in SPC	No. (%) of indications with RCT having OS as a primary endpoint in SPC
Hematological cancer	21	50.6	5 (24)	1 (5)	4 (19)	1 (5)
(a) Hematological malignancies	1	44.1	0 (0)	0 (0)	0 (0)	0 (0)
(b) Leukemia	10	49.6	3 (30)	1 (10)	1 (10)	1 (10)
(c) Lymphoma	9	54.2	2 (22)	0 (0)	3 (33)	0 (0)
(d) Multiple myeloma	1	35.6	0 (0)	0 (0)	0 (0)	0 (0)
Solid cancer	19	37.0	4 (21)	0 (0)	4 (21)	1 (5)
(a) Basal cell carcinoma	1	81.8	0 (0)	0 (0)	0 (0)	0 (0)
(b) Epithelial ovarian, fallopian tube, or primary peritoneal cancer	1	22.6	0 (0)	0 (0)	0 (0)	0 (0)
(c) Lung cancer	9	42.6	3 (33)	0 (0)	2 (22)	1 (11)
(d) Neuroblastoma	1	35.3	0 (0)	0 (0)	0 (0)	0 (0)
(e) Skin cancer	3	22.3	0 (0)	0 (0)	1 (33)	0 (0)
(f) Solid tumors	1	6.5	0 (0)	0 (0)	0 (0)	0 (0)
(g) Thyroid cancer	1	40.1	1 (100)	0 (0)	1 (100)	0 (0)
(h) Urothelial carcinoma	2	33.1	0 (0)	0 (0)	0 (0)	0 (0)
Overall	40	44.2	9 (22)	1 (2)	8 (20)	2 (5)

Abbreviations: OS, overall survival; RCT, randomized controlled trial; SPC, summary of product characteristics.

## 4 DISCUSSION

Our systematic review and quantitative synthesis comprising data from the EMA database shows that overall 79.9% of new approvals were supported by at least 1 RCT reported as the main study. This value dropped to 31.7% when considering RCTs with OS as the primary or co‐primary endpoint. However, in most cases, OS was included among the secondary endpoints. We observed relevant differences when considering solid and hematologic cancer as two different classes, with a higher frequency of RCTs (85.2% vs. 67.2%) and RCTs having OS as the primary endpoint (42.2% vs. 9.4%) in favor of solid cancer.

Further analysis of the indications not supported by RCTs at the time of approval (40 of 199) revealed a low frequency of post‐approval RCTs: After an average of 44.2 months from approval, in only nine cases (22%) at least one trial was reported in PubMed and only one (2%) had OS as a primary endpoint.

The main strength of our study is the systematic nature of the review and the consultation of the EMA database which publishes all clinical data used for the application for approval, in a standard and comprehensive format. This information represents the overall clinical evidence available at the approval of a new drug. Taken together, these two factors make our analysis robust, as a search based only on literature data on common scientific platforms may be influenced by unpublished studies or by publications reporting only partial data. The use of simple, objective, and categorical parameters such as randomized or non‐randomized design, and OS or non‐OS endpoint, made our results easily interpretable.

Our study has some limitations. In particular, the stage of the disease and the line of treatment were not considered, and the magnitude of the clinical effect reported as a result of the individual studies was not analyzed. Although these factors were beyond the scope of our research, they are important in defining the clinical value of a new drug. Given that all the drugs included in our analysis were approved, it is reasonable to assume that they have demonstrated sufficient clinical effect. A further limitation is the absence of a single study bias assessment, which could provide additional information about the validity of the clinical evidence. No distinctions were made between different non‐OS survival measures, which were overall classified in a single group, and between orphan status and conditional approvals. Finally, our study was intended to provide only a description of the clinical evidence, not including hypothesis testing between the differences observed between the different groups.

Our results are consistent with previous research by Davis et al., who showed that, among 68 indications of 48 cancer drugs approved by EMA from 2009 to 2013, 60 (88%) were supported by at least one RCT, 8 (12%) were approved on the basis of a single‐arm study, and 26% were supported by a pivotal study powered to evaluate OS as the primary outcome.^3^ A further study published by the same research group by Naci et al. examined the design characteristics, risk of bias, and reporting adequacy of pivotal RCTs of cancer drugs approved by EMA from 2014 to 2016. Among 32 approved cancer drugs based on 54 pivotal studies, 41 (76%) were RCTs and 13 (24%) were either non‐randomized studies or single‐arm studies. Only 10 RCTs (26%) measured OS as either a primary or co‐primary endpoint, and 19 RCTs (49%) were judged to be at high risk of bias for their primary outcome. Trials that evaluated OS were at a lower risk of bias than those that evaluated surrogate outcomes.^4^ The different result observed in our study (79% of indications supported by at least one RCT) is likely due to the inclusion of drugs approved in different time periods and only partially overlapping.

Grössmann et al. identified 134 different new anticancer drugs and indications approved by EMA between 2009 and 2016. A positive difference in median OS was associated with 76 licensed indications (55.5%); for 22 (16%) of them, a prolongation of more than 3 months could be observed. A positive difference in median PFS was observed in 90 indications (65.2%); 43 (31%) of them showed a positive difference of more than 3 months. For 37 indications (27%) no data were available for PFS and OS at the time of approval. In six indications (4.4%) a decrease in median OS was reported.^5^ A second study by the same group evaluated the number of new approvals that met the threshold for “meaningful clinical benefit” (MCB) according to the European Society for Medical Oncology Magnitude of Clinical Benefit Scale (ESMO‐MCBS). MCB was not met by most EMA‐approved cancer drugs, with limited evidence on the clinical benefit available at the time of approval in approximately half of the cases. The authors concluded that an approval status of an oncology drug may not confer a relevant health benefit for patients.^6^ Finally a third study by the same group, included originator anticancer drugs that were approved between 2009 and 2015, selecting those drugs with ambiguous benefit‐risk profiles, where no information or negative information on median OS was available at the time of approval. Among 102 approval studies, a negative difference in median OS or no information was available in 43 (42.2%) instances. After 3 years past EMA approval, there are still 29 therapies left (28.4%) with no or negative information (*n* = 24 [23.5%] and *n* = 5 [4.9%], respectively) regarding OS.^6^ Overall, the researches of Grössmann et al. provide interesting results complementary to those of our research, therefore mitigating its limitations. In particular, it is evident that in many cases, in addition to suboptimal design and use of endpoints, the magnitude of the clinical effect also appears limited, indicating that approval of a cancer drug is not synonymous with effective therapy.

Hatswell et al. systematically reviewed medicinal products approved by EMA and Food and Drug Administration (FDA) without an RCT from 1999 to 2014. A total of 44 indications were granted without RCT results by the EMA, including 19 indications for hematological malignancies and 9 for solid cancer.^8^ Our study found partially different results, due to the observation of two different periods. In particular, the absolute ratio of approvals without RCTs changed from approximately 2:1 to approximately 1:1 for hematological versus solid cancers, indicating an increasing adoption of alternative study designs for conducting pivotal trials in solid cancer setting over time.

Our results, showing a low availability of post‐registration RCTs for indications that had no RCTs at approval, are in agreement with an analysis of 54 approvals made by the United States’ FDA from 2008 to 2012. A total of 36 drugs (67%) were approved on the basis of a surrogate endpoint. After a median follow‐up of 4.4 years, five drugs were subsequently shown to improve OS in randomized studies, 18 drugs failed to improve OS as primary or secondary outcomes, and 13 (24%) drugs continue to have unknown survival effects, meaning they remain untested or they have no reported survival results as primary or secondary outcome.^9^

Our results show that about one of five approved drugs was based on uncontrolled studies and that only 25% of these cases had at least one randomized study after an average time of about 3.6 years from approval. This represents a major concern, given that RCTs still represent the highest level of evidence‐based medicine, and that RCTs are the gold standard when the aim of the research is to evaluate the intended effect of an intervention.^23^, ^24^, ^25^ Randomization was proposed as the key element in defining added value drugs in an analysis that assessed anticancer drugs for hematological malignancies approved by EMA from 1995 to 1996. No added value was established for about two‐thirds of the drugs, primarily due to methodological concerns related to study design (absence of randomization) and endpoint robustness.^26^ However, even when randomization takes place, it is not necessarily a guarantee of higher quality, for example, of 95 anticancer drugs approved by the FDA from 2013 to 2018, 16 (17%) were based on RCTs with suboptimal control arms. When categorized by the nature of suboptimal control, 4 (25%) trials omitted active treatment in the control arm by limiting investigator's choice, 11 (63%) trials omitted active treatment in the control arm by using a control agent known to be inferior to other available agents or not allowing combinations, and 1 (13%) trial used a previously used treatment in the control arm with a known lack of benefit associated with re‐exposure. Although anticancer drug approvals are increasing, a proportion of these drugs are reaching the market without proven superiority to what is considered the standard of care at the time of patient enrollment in pivotal trials.^27^

We found that far fewer than half of the studies were blinded. This represents a possible source of bias. If patients are not blinded to their allocated treatment, then its awareness may influence their responses to the intervention and their reporting. Commonly, patients assume that the new intervention will be more beneficial than the control or standard treatment. Compared with clinical event outcomes, patient‐rated outcomes (e.g., QoL, pain, and discomfort) are particularly sensitive to patients’ knowledge of the intervention to which they have been allocated. Similarly, if investigators are aware of the patients’ study treatment, their knowledge may influence, first, their management of the patient and, second, their classification of responses and events.^28^

Overall, our observations and previously discussed evidence indicate the suboptimal quality of clinical evidence supporting the approval of new anticancer drugs in Europe, in terms of study design, efficacy endpoints, and extent of clinical benefit demonstrated. Furthermore, in most cases, the absence of solid evidence at approval persists even after several years. Therefore, public regulators should further encourage and facilitate the conduct of randomized pivotal trials capable of providing the highest level of evidence. This issue was recently and brilliantly discussed by Naci et al., who proposed a set of five principles to promote the production of high‐quality CE to support decision making, briefly: head‐to‐head comparisons to be routinely reported on product labels; more selective use of expedited programs, including well‐designed evidence‐generation plans to be conducted in the post‐marketing period; more routine use of active‐comparator RCTs; network meta‐analyses to be performed within each therapeutic area, and higher harmonization in the methods of registration studies; CE data to be a crucial factor in pricing and payment decisions.^29^

The agenda for further research to clarify the open points is related to the limitations of our study, in particular, it will be useful to systematically evaluate the magnitude of the clinical effect in relation to the specific conditions and the risk of bias of the individual studies. In addition, we propose further standardization and harmonization in reporting study results by EMA. In fact, this research was conducted by manually consulting the specific EPAR product pages and extracting the searched data. This takes a long time and creates potential sources of human error. Therefore, an aggregated database would be useful to further foster research in this field and make clinical evidence supporting drug use in the EU even more transparent.

## 5 CONCLUSION

Our systematic review of the indications for the treatment of solid or hematological tumors approved by EMA from 2010 to 2019 shows that approximately four of five new approvals were supported by at least 1 RCT at the time of approval. The quality of these RCTs was heterogeneous and a substantial proportion of new approvals that are not based on RCTs is lacking post‐marketing RCTs. Future efforts should focus on further improving the availability of data from preferably blinded RCTs reporting on OS and QoL at the time of approval of a new cancer drug.

## DISCLOSURE

AF is employed in the pharmaceutical industry but without conflicts of interest for this work. TS received grants from the Federal German Ministry of Health, the Kurt Goldstein Institute, and the Michael J. Fox Foundation that were not related to this study. FF, FM, AS, MB, none declared.

## AUTHOR CONTRIBUTION

All authors are qualified for authorship. Authors’ contributions: AF involved in conception, development, data collection and analysis, writing, and final approval of the manuscript. FM carried out data collection, critical review, and approval of the manuscript. FF, AS, and MB involved in interpretation of data, critical review, and approval of the manuscript. TS carried out conception, development, interpretation of data, review, and approval of the manuscript. All authors are accountable for all aspects of the work in ensuring accuracy or integrity.

## ETHICS APPROVAL STATEMENT

Not applicable.

## PATIENT CONSENT STATEMENT

Not applicable.

## PERMISSION TO REPRODUCE MATERIAL FROM OTHER SOURCES

Not applicable.

## Data Availability

No additional data available.

## References

[1] World Health Organization . Fact sheet, cancer. https://www.who.int/news‐room/fact‐sheets/detail/cancer. Accessed January 24, 2021.

[2] https://www.ema.europa.eu/en/medicines/download‐medicine‐data. Accessed January 24, 2021.

[3] Davis C , Naci H , Gurpinar E , et al. Availability of evidence of benefits on overall survival and quality of life of cancer drugs approved by European Medicines Agency: retrospective cohort study of drug approvals 2009–13. BMJ. 2017;359:j4530. 28978555 10.1136/bmj.j4530 PMC5627352

[4] Naci H , Davis C , Savović J , et al. Design characteristics, risk of bias, and reporting of randomised controlled trials supporting approvals of cancer drugs by European Medicines Agency, 2014–16: cross sectional analysis. BMJ. 2019;366:l5221. 31533922 10.1136/bmj.l5221 PMC6749182

[5] Grössmann N , Wild C . Between January 2009 and April 2016, 134 novel anticancer therapies were approved: what is the level of knowledge concerning the clinical benefit at the time of approval? ESMO Open. 2017;1:e000125. 28848662 10.1136/esmoopen-2016-000125 PMC5548976

[6] Grössmann N , Del Paggio JC , Wolf S , et al. Five years of EMA‐approved systemic cancer therapies for solid tumours‐a comparison of two thresholds for meaningful clinical benefit. Eur J Cancer. 2017;82:66‐71. 28648700 10.1016/j.ejca.2017.05.029

[7] Grössmann N , Robausch M , Rosian K , et al. Monitoring evidence on overall survival benefits of anticancer drugs approved by the European Medicines Agency between 2009 and 2015. Eur J Cancer. 2019;110:1‐7. 30735832 10.1016/j.ejca.2018.12.026

[8] Hatswell AJ , Baio G , Berlin JA , et al. Regulatory approval of pharmaceuticals without a randomised controlled study: analysis of EMA and FDA approvals 1999–2014. BMJ Open. 2016;6:e011666. 10.1136/bmjopen-2016-011666 PMC4932294 27363818

[9] Kim C , Prasad V . Cancer drugs approved on the basis of a surrogate end point and subsequent overall survival: an analysis of 5 years of US Food and Drug Administration approvals. JAMA Intern Med. 2015;175:1992‐1994. 26502403 10.1001/jamainternmed.2015.5868

[10] Spieth PM , Kubasch AS , Penzlin AI , et al. Randomized controlled trials—a matter of design. Neuropsychiatr Dis Treat. 2016;12:1341‐1349. 27354804 10.2147/NDT.S101938 PMC4910682

[11] Koretz RL . Assessing the evidence in evidence‐based medicine. Nutr Clin Pract. 2019;34:60‐72. 30570169 10.1002/ncp.10227

[12] Sorenson C , Naci H , Cylus J , et al. Evidence of comparative efficacy should have a formal role in European drug approvals. BMJ. 2011;343:d4849. 21896610 10.1136/bmj.d4849

[13] Tannock IF , Amir E , Booth CM , et al. Relevance of randomised controlled trials in oncology. Lancet Oncol. 2016;17:e560‐e567. 27924754 10.1016/S1470-2045(16)30572-1

[14] Broglio KR , Berry DA . Detecting an overall survival benefit that is derived from progression‐free survival. J Natl Cancer Inst. 2009;101:1642‐1649. 19903805 10.1093/jnci/djp369 PMC4137232

[15] Fleming TR , Rothmann MD , Lu HL . Issues in using progression‐free survival when evaluating oncology products. J Clin Oncol. 2009;27:2874‐2880. 19414672 10.1200/JCO.2008.20.4107 PMC2698020

[16] Naci H , Davis C . Inappropriate use of progression‐free survival in cancer drug approvals. BMJ. 2020;368:m770. 32156802 10.1136/bmj.m770

[17] Saturni S , Bellini F , Braido F , et al. Randomized controlled trials and real life studies. Approaches and methodologies: a clinical point of view. Pulm Pharmacol Ther. 2014;27:129‐138. 24468677 10.1016/j.pupt.2014.01.005

[18] Booth CM , Eisenhauer EA . Progression‐free survival: meaningful or simply measurable? J Clin Oncol. 2012;30:1030‐1033. 22370321 10.1200/JCO.2011.38.7571

[19] Chen EY , Joshi SK , Tran A , et al. Estimation of study time reduction using surrogate end points rather than overall survival in oncology clinical trials. JAMA Intern Med. 2019;179:642‐647. 30933235 10.1001/jamainternmed.2018.8351 PMC6503556

[20] EMA/CHMP/205/95 Rev.5. Committee for Medicinal Products for Human Use (CHMP) . Guideline on the evaluation of anticancer medicinal products in man. https://www.ema.europa.eu/en/documents/scientific‐guideline/guideline‐evaluation‐anticancer‐medicinal‐products‐man‐revision‐5_en.pdf. Accessed December 13, 2020.

[21] Moher D , Liberati A , Tetzlaff J , et al. Preferred reporting items for systematic reviews and meta‐analyses: the PRISMA statement. Int J Surg. 2010;8:336‐341. 20171303 10.1016/j.ijsu.2010.02.007

[22] https://www.ema.europa.eu/en/medicines. Accessed December 13, 2020.

[23] Bosdriesz JR , Stel VS , van Diepen M , et al. Evidence‐based medicine‐When observational studies are better than randomized controlled trials. Nephrology. 2020;25:737‐743. 32542836 10.1111/nep.13742 PMC7540602

[24] Djulbegovic B , Guyatt GH . Progress in evidence‐based medicine: a quarter century on. Lancet. 2017;390:415‐423. 28215660 10.1016/S0140-6736(16)31592-6

[25] Vere J , Gibson B . Evidence‐based medicine as science. J Eval Clin Pract. 2019;25:997‐1002. 30575209 10.1111/jep.13090

[26] Bertele’ V , Banzi R , Capasso F , et al. Haematological anticancer drugs in Europe: any added value at the time of approval? Eur J Clin Pharmacol. 2007;63:713‐719. 17530236 10.1007/s00228-007-0296-2

[27] Hilal T , Sonbol MB , Prasad V . Analysis of control arm quality in randomized clinical trials leading to anticancer drug approval by the us food and drug administration. JAMA Oncol. 2019;5:887‐892. 31046071 10.1001/jamaoncol.2019.0167 PMC6499129

[28] Forder PM , Gebski VJ , Keech AC . Allocation concealment and blinding: when ignorance is bliss. Med J Aust. 2005;182:87‐89. 15651970 10.5694/j.1326-5377.2005.tb06584.x

[29] Naci H , Salcher‐Konrad M , Kesselheim AS , et al. Generating comparative evidence on new drugs and devices before approval. Lancet. 2020;395:986‐997. 32199486 10.1016/S0140-6736(19)33178-2

